# Could electrohypersensitivity be a specific form of high sensory processing sensitivity?

**DOI:** 10.3389/fpubh.2025.1550427

**Published:** 2025-02-28

**Authors:** Jimmy Bordarie, Maryse Ledent, Mael Dieudonné, Frédéric Choisay, Eva De Clercq

**Affiliations:** ^1^QUALIPSY (UR1901), Psychology Department, University of Tours, Tours, France; ^2^Chemical and Physical Health Risks, Sciensano, Brussels, Belgium; ^3^Centre Max Weber, Lyon, France

**Keywords:** electrohypersensitivity, sensory processing sensitivity, electromagnetic fields, risk perception, absorption, anxio-depressive disorders

## Abstract

**Introduction:**

Electrohypersensitivity (EHS) refers to a syndrome in which individuals claim to suffer from a variety of symptoms that they attribute to electromagnetic fields. The characteristics of this specific hypersensitivity, particularly in terms of symptoms, are similar to those associated with high sensory processing sensitivity (HSPS). This article raises the question of the superposition of these two types of sensitivity and investigates the existence of a link between the two.

**Methods:**

Participants (*n* = 100) completed a questionnaire measuring EHS and HSPS, as well as absorption, risk perception and avoidance strategies related to electromagnetic fields, and anxiety and depressive disorders.

**Results:**

They showed an overrepresentation of highly sensitive people within the electrohypersensitive group. Furthermore, the results showed differences in terms of anxiety-depressive symptomatology and cognitive strategies (risk perception and avoidance strategies).

**Discussion:**

The article discusses these results in the light of the literature and suggests avenues for future research and ways to help highly sensitive people, whether wor not this condition is considered to be caused by electromagnetic radiation.

## 1 Introduction

Electrohypersensitivity (EHS) refers to a syndrome in which individuals claim to suffer from a variety of symptoms that they attribute to electromagnetic field (EMF) sources, in the absence of validated clinical or biological evidence to explain these symptoms (1, 2). The WHO classified this reported sensitivity as idiopathic environmental intolerance (IEI), which includes syndromes in which individuals describe symptoms related to environmental exposures in the absence of overt clinical abnormalities (3). There are many examples of such syndromes, such as sick building syndrome, sensitivity to multiple physical, chemical and biological agents, etc. Among physical agents, EMFs give rise to the IEI attributed to EMF (IEI-EMF) syndrome. The incriminated sources are increasingly widespread in our modern societies, e.g., telecommunication antennas, mobile phones, WI-FI routers, high-voltage power lines, computer screens, electrical appliances, etc. In a certain number of cases, IEI-EMF would not be an isolated high sensitivity in the sense that several authors claimed that a proportion of IEI-EMF sufferers also report other sensitivities (e.g., multiple chemical sensitivities) (1, 4). Studies on the prevalence of EHS report results ranging from 0.7 to 13.3% (1). Part of this variability can be explained by the lack of objective criteria to study it. In this context, prevalence is very often assessed by a single yes/no question asking if the person is highly sensitive (5). These authors hypothesized that there is great heterogeneity in the characteristics of people who self-report EHS. As inclusion criteria in empirical studies, Szemerszky et al. (5) suggested supplementing with additional questions, such as onset of symptoms and negative impact on daily life.

The literature is rich, and the methodologies developed to investigate the EMF hypothesis in EHS are numerous, such as observational studies [e.g., (6, 7)], ecological momentary assessment studies [e.g., (8, 9)], provocation studies [e.g., (10–14)] or intervention studies [e.g., (15–17)]. Despite a growing body of literature and these different methodological approaches, the conclusions to date tend to reject the existence of valid clinical or biological evidence linking these symptoms to EMFs (1, 18). The results of the meta-analysis of provocation studies by Schmiedchen et al. (19) also support this view of an unlikely effect of EMF exposure as an explanation for these symptoms but point to methodological limitations in the published studies. Anses (1) encouraged the continuation of such experiments with innovative protocols that take the limitations into account. In addition to a direct link between EMF exposure and symptoms (the so-called EMF hypothesis), several hypotheses have emerged to explain the symptoms, such as the cognitive and attributional hypotheses (20). In the cognitive hypothesis, a nocebo effect has been proposed several times (12, 21, 22). The nocebo effect corresponds to the effect that occurs when one (consciously or unconsciously) expects negative consequences from certain factors (e.g., EMFs, due to their ubiquity and the controversies they generate in society, may lead some individuals to expect that they will have a negative impact on them, their health and/or their life in general). However, this hypothesis cannot be fully satisfactory as it can only be applied to people who *a priori* consider EMF as a source of health hazards. An attributive hypothesis has also been proposed in a part of the literature that focuses on qualitative approaches and is interested in the trajectories of people with EHS who perceive themselves as such. These studies have shown that the same symptoms can be pre-existing and attributed to EMFs, while there was no fear before (23–25). As suggested by Dieudonné (24), the nocebo effect can occur when the association is established.

The different hypotheses led to controversies in the literature (20, 26), while Van den Bergh et al. (27) suggested that IEIs should share the same origin, a causal belief. These authors proposed a comprehensive model of IEI (27). The model is based on the principles of recent Bayesian predictive coding models of brain function, which treat symptom perception as active inference. On this basis, Haanes et al. (28) recommend using the term “symptoms associated with environmental factors” (SAEF) to describe these conditions, which is consistent with the perceptual elements that appear to underlie them. In the comprehensive model, causal belief would lead to anticipation and nocebo effects in relation to the perceived exposure, while symptoms would lead to belief validation (attribution) (27). This could then be explained by the existence of dispositional variables, i.e., variables such as personality or temperamental traits, which could then make it possible to understand either the occurrence of these symptoms or the tendency to attribute them to EMFs. For example, holistic thinking style (i.e., the tendency toward spirituality, holistic health beliefs and connection with nature) and somatic symptom distress would be the most important factors at play in IEI-EMF (5). Absorption (i.e., the ability to become deeply involved in a sensory and imaginary event while completely ignoring other stimuli) (29) is higher in people reporting IEI-EMF (27, 30, 31). This personality trait is known to enhance symptom attributions to EMF as well as somatosensory amplification, which is also hypothesized to contribute to EHS (31, 32). In addition, the literature also confirms that people with EHS tend to feel inferior to others and feel uncomfortable in their social relationships (33, 34). They would also struggle more to maintain a certain level of self-esteem, have a more altered self-image and be more vulnerable (35). Finally, they would feel more anger and hostility toward others (34). In addition, Anses (1) suggests that the existence of a temperamental trait, high sensory processing sensitivity, and its biological basis, at least in part, could be a way of thinking about a possible common factor between EHS and other medically unexplained disorders or syndromes that deserves to be explored. However, this hypothesis does not appear to have been the subject of any study to date.

Sensory processing sensitivity (SPS) is a temperamental trait (36) that is considered innate and stable (37), affecting over 30% of the population (38). Individuals with higher sensory sensitivity than their peers (39) are then described as highly sensitive and could easily adapt to environmental stressors (40, 41). SPS is a multidimensional construct in which three (i.e., Ease of Excitation [EOE], Low Sensory Threshold [LST], AEsthetic Sensitivity [AES] (42)) or four (controlled harm avoidance [CHA] is added to the previous ones (43, 44)) components coexist. The EOE refers to the feeling of being easily overwhelmed by stimuli, the LST refers to the existence of a particularly low threshold of sensitivity, the AES refers to a sensitivity to fine distinctions related to, for example, art, and the CHA seems to be a strategy used by people to avoid certain stimuli as much as possible, for example by being conscientious. Each component then plays a different role, sometimes protective, sometimes vulnerable [e.g., (45)]. Although not a pathology, high sensory processing sensitivity (HSPS) is often the cause of certain psychopathological disorders, particularly those related to perceived stress (46–48), depression (49, 50) and anxiety (45, 51). Highly sensitive people also seem to be more prone to burnout (52, 53) or to report poorer health (48). The different components then appear to play specific roles. For example, EOE and LST generally tend to increase levels of distress related to general anxiety and depression (54, 55). Some studies suggest that the propensity to develop a depressive state could be mainly related to EOE (54) or LST (56). On the contrary, other studies have shown that AES is rarely—and sometimes negatively—correlated with depressive symptomatology (45) but is proportional to anxiety in general (42) (54–56).

To our knowledge, and as already pointed out in Anses (1), no study has been conducted on the existence of a link between high (sensory processing) sensitivity and the occurrence of syndromes, symptoms related to the environment or IEIs, such as EHS or multiple chemical sensitivity (MCS). However, highly sensitive people (HSP) and EHS share certain characteristics, in particular their health outcomes. In general, EHS is associated with greater psychiatric comorbidity and poorer mental health (57), whereas HSPS is associated with poorer general health (48). Indeed, like HSPS, EHS is correlated with depressive affect (58), anxiety symptoms and higher levels of stress (33, 59). Other findings suggest an association between EHS and difficulties with positive affect, anxiety, doubt and phobias (33) or phobic anxiety (34). People with EHS feel uncomfortable in their social relationships, whereas highly sensitive people (HSP) report more social phobia (60) and agoraphobic avoidance (61). This is a risk given that negative affect has been shown to play an important role in attributing symptoms to environmental causes (27). Furthermore, EHS is also associated with high levels of neurasthenia (a persistent state of despondency accompanied by sadness) (59) and a greater tendency to neuroticism, which is a personality trait characterized by a persistent tendency to experience negative emotions (62). HSPS is also positively associated with neuroticism. It is also positively correlated with introversion (personality trait characterized by psychic energy on the subject himself, attentive to his inner world rather than the outside world) (63), and it is negatively correlated with extraversion (personality trait characterized by great ease in establishing contact with those around him, who readily express their feelings) (38, 42, 54) (64–66).

## 2 Aims and hypotheses

Since the current literature does not allow us to conclude that there is a relationship between exposure to EMF sources and the symptoms reported by EHS sufferers, there are other scientific avenues for understanding the symptoms of people who initially attribute them to EMFs. Among these avenues, the possible influence of dispositional variables cannot be ignored, given the literature on the subject. In particular, the case of sensory processing sensitivity has attracted our attention since Anses (1) invited researchers to explore this avenue. Indeed, it tends to produce health effects similar to those attributed by EHS people to EMFs. In this study, we investigate the nature of the relationship between HSPS and EHS. The parallelism of the consequences for the health of people affected by HSPS and EHS raises an essential question: are these clinical pictures variants of the same trait, or do they each constitute a distinct entity? The existence of high sensitivity as a personality trait, as well as its biological and neurological underpinnings, provide “food for thought about a possible factor common to EHS and other medically unexplained disorders or syndromes that deserves to be studied” (1). As this hypothesis has not been investigated to date, this exploratory study proposes to address it by attempting to answer the following hypotheses:

*Hypothesis* 1: Self-assessed EHS individuals have higher sensory processing sensitivity scores than others.

*Hypothesis* 2: Self-assessed EHS individuals have higher scores on EOE, LST (which may explain some of their symptoms) and CHA (which may be related to their tendency to engage in EMF-specific avoidance strategies).

*Hypothesis* 3: Highly sensitive people (HSP) are overrepresented in the self-assessed EHS group.

In addition, anxiety and depressive disorders, perception and behaviors are examined as part of the following hypotheses, allowing us to explore the predominant traits:

*Hypothesis* 4: Self-assessed EHS individuals have higher absorption scores than other groups.

*Hypothesis* 5: Self-assessed EHS individuals have a higher risk perception, daily avoidance scores, depression and anxiety than other groups.

Finally, we also aim to check the relevance of the criteria of Szemerszky et al. (5):

*Hypothesis* 6: The criteria of Szemerszky et al. lead to the distinction of two groups (EHS++ and EHS+) within the group of people who assess themselves as EHS.

*Hypothesis* 7: EHS++ have higher scores (HSPS, absorption, risk perception, avoidance strategy, depression and anxiety) than EHS+.

## 3 Methods

We report the results of the ExpoComm and ENVI-EHS projects which were conducted between 2018 and 2022. In the ExpoComm project, a provocation protocol was co-designed with EHS people (67) and then used with participants to test its effectiveness in detecting real or simulated exposure situations. In order to get a larger sample, the ENVI-EHS project was launched as an extension of ExpoComm for another year.

### 3.1 Procedure and recruitment

People were recruited through information on the partners’ internet and intranet websites and environmental and EHS associations, a press release and a press conference in December 2020 to present the project, posters presenting the project and distributed in local shops and institutes, and word of mouth. Four groups were initially formed at the inclusion stage: (1) EHS (people who assessed themselves as EHS), (2) EHS? (people who questioned their sensitivity), (3) SNS (people with non-specific symptoms of unknown origin, like those reported by EHS people, but not attributed to EMF), and (4) nonEHS (people who did not identify or assess themselves as IEI-EMF and did not report any symptoms).

During the initial contact, volunteers received general information about the project and an information dossier. If they were interested in participating, they were invited to attend the third step of the protocol (67), namely a habituation session. Both EHS (1 and 2) and nonEHS (3 and 4) groups attended this session, which aimed to present the test environment and to experience the test conditions; the latter was conducted in the open field and was particularly important for the EHS/EHS volunteers to test the accuracy of the exposure system and condition on their sensitivity. Prior to this session, volunteers were given the Informed Consent Form (ICF) and were first asked to fulfil the questionnaire, as described below. The ICF was signed after the presentation of the test environment and the completed questionnaire was given to the researcher. Data collection took place between May 2019 and April 2022.

### 3.2 Measurements

#### 3.2.1 Electrohypersensitivity

In addition to the self-definition of their EHS at inclusion (which resulted in the four groups mentioned above), EHS volunteers were assigned an EHS score based on Szemerszky et al. (5). This score was calculated by summing the EHS volunteers’ responses to the questionnaire regarding:

- EMF interference with daily life, from 0 (not at all) to 4 (completely).
- The sum of symptoms reported in the Environmental Hypersensitivity Symptom Inventory (EHSI) attributed to EMF exposure [translated from Nordin et al. (68)] was divided into four parts: 0 (no symptoms), 1 (between 1 and 8 symptoms), 2 (between 9 and 17 symptoms), and 3 (more than or equal to 18 symptoms).

The EHS score could range from 0 to 7; EHS individuals with an EHS score of 5 or higher are considered as EHS++ and the others as EHS+.

#### 3.2.2 Sensory processing sensitivity

Sensory processing sensitivity was measured using the French version of the HSPS scale (36). The psychometric properties of this scale have been confirmed several times (69). It consists of 27 items measuring individuals’ cognitive and emotional responses to various environmental stimuli. Responses are scored on a 7-point Likert scale. The study was conducted prior to the validation and publication of the French translation of the scale (HSPS-FR) (43). However, the two versions are very close. We then used the model highlighted in the French adaptation, which proposes 4 dimensions: (1) EOE with items 1, 3, 4, 11, 13, 14, 16, 20, 21, 26, 27, (2) LST with items 5, 6, 7, 9, 18, 19, 23, 25, (3) AES with items 2, 8, 10, 15, 22 and (4) CHA with items 12, 17, 24.

Using a categorical approach, we can consider three groups according to their sensory processing sensitivity scores (38). Hyposensitive people, whose scores are strictly below 113 and generally represent around 30% of the population. Highly sensitive people, whose scores are strictly above 137, also represent about 30% of the population. Finally, people with sensitivity scores between 113 and 137 represent around 40% of the population and are considered to have average sensitivity.

#### 3.2.3 Absorption

Absorption was measured using the multidimensional Tellegen Absorption Scale (TAS) (29). It consists of a 34-item true/false scale that assesses imaginative involvement and the tendency to become mentally absorbed in everyday activities. It measures “openness to self-absorbing and self-modifying experiences” (29) and is generally related to the imaginative involvement facets of openness (i.e., fantasy, esthetics and feelings) (70). Responses are scored on a dichotomous true (1)/false (0) scale. Tellegen (71) reported an internal reliability of *r* = 0.88 and a 30-day test–retest reliability of *r* = 0.91. A global TAS score is calculated by summing the responses.

#### 3.2.4 Anxiety and depressive disorders

A score for depression was calculated based on the nine items of the PHQ-9 scale (72). For anxiety, a score was calculated based on the seven items of the GAD-7 scale (73). In both scales, the frequency of the items was rated on a scale from 0 (“never”) to 3 (“almost every day”). Scores were calculated by summing the responses.

#### 3.2.5 Risk perception and avoidance strategies

To assess risk perception, participants rated the hazardousness of nine EMF sources on a scale from 0 (no risk) to 10 (maximum risk). The scale includes sources such as Wi-Fi access points, base stations, phones, computers, household appliances, power lines and smart meters. A risk perception score was calculated by averaging the scores assigned to each source on a scale of 0 to 10.

To assess EMF exposure avoidance strategies, participants listed the actions they had taken, specifying whether each had been taken within the last month (scored as 1 point) or more than a month ago (scored as 2 points). If a strategy was not applied, no point was awarded. The list included 15 actions, such as reducing the use of devices, asking others to turn off devices, avoiding certain places or people, changing the home or work environment, and wearing protective clothing. The cumulative score for all strategies was then divided into five quantile-based groups: 0 (no strategies implemented), 1 (scores of 1–2), 2 (scores of 3–7), 3 (scores of 8–13) and 4 (scores of 14 or more), which were used in the analyses.

### 3.3 Sample

The study included 100 adults. However, one participant was excluded from the analyses due to a high proportion of non-responses to the various items. The characteristics are shown in Table 1. The sample was 53% female and 47% male. The mean age was 48.1 years (SD = 13.4) and ranged from 22 to 76 years. Based on self-report of sensitivity (EHS self-report), four groups were formed, including 33 EHS, 14 EHS?, 19 SNS and 35 nEHS. Using the criteria of Szemerszky et al. to divide the EHS group (EHS-criteria), a group of 23 EHS++ and a group of 10 EHS+ were formed.

**Table 1:** Characteristics of the sample.

	First classification according to self-assessment				EHS classification Szemerszky et al. (5)	
	EHS	EHS?	SNS	nEHS	EHS++	EHS+
	N	N	N	N	N	N
Total	33	13	19	35	23	10
Gender						
Female	16	4	13	20	11	5
Male	17	9	6	15	12	5
Status						
Single	14	4	6	11	11	3
Married or cohabiting	11	8	12	20	5	6
Divorced or separated	8	1	1	3	7	1
Widowed	0	0	0	1	0	0
Education level						
Primary school	1	1	0	0	0	1
Lower secondary school	3	0	0	0	3	0
Higher secondary school	7	3	2	3	4	3
High school (short) (<3 years)	11	1	7	8	8	3
High school (long) (>3 years)	11	8	10	18	8	3
Post-university	0	0	0	6	0	0
Occupational status						
Student	2	0	1	3	2	0
Incapacity to work	5	1	0	0	5	0
Pension	4	3	1	3	1	3
Unemployed	3	1	0	2	3	0
Trainings	2	0	0	0	1	1
Illness/career break	4	0	0	2	3	1
Full-time	7	6	14	16	3	4
Part-time	6	2	3	9	5	1

### 3.4 Statistical analyses

Data analysis was performed using JASP 2023 (version 0.17.1) and STATA (version 17). Correlations and Chi2 were performed. Student’s t-tests, Anova and Bonferroni’s post-hoc (*p*-Bf) test were used for HSPS-FR scores, which were normally distributed. For absorption, anxiety and depressive disorders, risk perception and avoidance strategies, we used non-parametric tests, such as Kruskal-Wallis test and Dunn’s post-hoc test (*p*-Dunn) to compare the four baseline groups and the Wilcoxon rank sum test was used to compare the two EHS groups formed according to the criteria of Szemerszky et al. (5).

## 4 Results

### 4.1 Sensory processing sensitivity

Of the 87 valid questionnaires, the minimum score for the HSPS was 39 and the maximum score was 181. The mean total score was 115.91 (SD = 29.49). Following the classification of Lionetti et al. (38), approximately 43.7% of the sample (*n* = 38) reported low HSPS (total score strictly below 113), 32.2% (*n* = 28) reported moderate HSPS (score between 113 and 137), and 24.1% of the sample (*n* = 21) reported high HSPS (score strictly above 137). Scores for each scales according to groups [means and standard deviations (SD) for HSPS; median and interquartile range (IQR) for other scales] are reported for Table 2.

**Table 2:** Scores for each scales according to groups (means and standard deviations (SD) for HSPS; median and interquartile range (IQR) for other scales).

	First classification according to self-assessment								EHS classification according to Szemerszky et al. (5)			
	EHS+ (*n* = 27)		EHS? (*n* = 12)		SNS (*n* = 18)		nEHS (*n* = 30)		EHS++ (*n* = 18)		EHS+ (*n* = 9)	
	Means	SD	Means	SD	Means	SD	Means	SD	Means	SD	Means	SD
HSPS (total)	129.81	24.47	119.42	29.33	114.11	18.73	103.07	33.95	128.17	27.12	133.11	19.10
EOE	48.74	11.88	46.58	14.10	45.83	8.91	39.67	15.36	47.94	13.47	50.33	8.31
LST	39.74	8.47	36.25	12.02	32.17	9.36	29.40	12.70	39.39	8.73	40.44	8.37
AES	24.78	5.33	21.58	5.47	20.00	4.49	20.13	6.25	24.78	6.01	24.78	3.96
CHA	16.56	3.38	15.00	1.81	16.11	3.23	13.87	3.76	16.06	3.92	17.56	1.67
	Median	IQR	Median	IQR	Median	IQR	Median	IQR	Median	IQR	Median	IQR
Absorption (total) (*n* = 21)	14	11	12	8	13	4	11	7	14	12	13.5	12
Anxiety (*n* = 29)	7	10	4	4	3	5	2	3	8.5	10	5	5
Depression (*n* = 32)	8	8	5.5	3	5	6	2.5	4	9.5	9	6	8
Risk perception (*n* = 33)	6.22	2.38	4.13	3.25	3.67	3.57	2.11	3.67	7.44	2.11	5.72	3.07
Avoidance strategies (*n* = 33)	3	3	2	2	0	0	0	0	3	2	1	1

The Chi2 test showed an overrepresentation of highly sensitive individuals in the self-assessed EHS group (Fisher’s exact: *p* = 0.01). We also found differences according to gender (*t* = 2.516; *p* = 0.014; Table 3) but not according to age categories.

**Table 3:** T test for HSPS according to sex.

Cases	Obs	Mean	Standard error	Standard deviation	95% interval confidence		t	df	*p*
Female	46	123.196	4.045	27.437	115.048	131.343	2.516	85	0.0069
Male	41	107.732	4.668	29.887	98.298	117.165			
Combined	87	115.908	3.162	29.490	109.623	122.193			
Diff		15.464	6.146		3.244	27.684			

There was a significant difference between the four groups (*F* = 4.46; *p* = 0.006) and more specifically the difference appeared between the self-assessed EHS group and the nonEHS group (*t* = 3.619; *p*-Bf = 0.003): the first ones obtained higher SPS scores than the nonEHS group. There were also significant differences for three of the HSPS components between EHS and nonEHS: LST (*F* = 4.73; *p* = 0.004; *p-*Bf = 0.003), CHA (*F* = 3.52; *p* = 0.019; *p-*Bf = 0.019) and AES (*F* = 4.15; *p* = 0.009; *p-*Bf = 0.013). There was also a significant difference for AES between EHS and SNS (*p-*Bf = 0.034; Table 4). For EOE, we can mention a tendency (*F* = 2.49; *p* = 0.066).

**Table 4:** HSPS scores and its components according to EHS self-assessment and EHS-criteria.

	First classification according to self-assessment (ANOVA)					EHS classification according to Szemerszky et al. (5) (Student’s *t*-test)		
	Sum of Squares	df	Mean Square	F	*p*	*t*	df	*p*
EHS-HSPS	10374.63	3	10374.63	4.46	0.006	−0.4876	25	0.630
Residuals	64414.64	83	64414.64					
EHS-EOE	1266.55	3	1266.55	2.49	0.066	−0.4853	25	0.632
Residuals	14049.27	83	14049.27					
EHS-LST	1644.47	3	1644.47	4.73	0.004	−0.3000	25	0.767
Residuals	9619.14	83	9619.14					
EHS-AES	381.39	3	381.39	4.15	0.009	0.0000	25	1.000
Residuals	2541.05	83	2541.05					
EHS-CHA	117.08	3	117.08	3.52	0.019	−1.0917	25	0.285
Residuals	919.91	83	919.91					

Using the criteria of Szemerszky et al. (5), the T-test showed that there was no significant difference between EHS++ and EHS+ for the HSPS score (Table 4).

### 4.2 Absorption

Among the 94 valid questionnaires, the minimum score for absorption was 1 and the maximum was 37. The median score was 13 (IQR = 7). The non-parametric Kruskal-Wallis test was not significant (X2(3) = 3.009; *p* = 0.3902; Table 5). No significant difference was observed neither by sex (z = 1.63; *p* = 0.10) nor by age categories (X^2^(4) = 2.89; *p* = 0.58).

**Table 5:** Comparison of group scores according to categorization into four groups (EHS self-assessment) and two groups (EHS-criteria).

		First classification according to self-assessment Kruskal–Wallis equality-of-populations rank test						EHS classification according to Szemerszky et al. (5) (Wilcoxon rank sum test)	
		EHS	EHS?	SNS	nEHS	Chi2 (3)	*p*	z	*p*
Absorption	Participants	29	13	19	33	3.009	0.3902	0.970	0.3300
	Rank-sum	1574.00	547.00	908.00	1436.00				
Anxiety	Participants	31	13	19	34	19.635	**0.0002**	2.245	**0.0247**
	Rank-sum	2021.50	674.50	885.50	1171.50				
Depression	Participants	32	12	19	34	3.286	**0.0001**	1.896	0.0579
	Rank-sum	2022.00	638.00	1042.50	1050.50				
Risk perception	Participants	33	13	19	35	31.431	**0.0001**	2.567	**0.0103**
	Rank-sum	2398.00	634.50	814.00	1203.50				
Avoidance strategies	Participants	33	13	19	35	47.611	**0.0001**	4.375	**<0.001**
	Rank-sum	2490.50	809.50	631.50	1118.50				

The significant values in bold.

Using the criteria of Szemerszky et al. (5), the Wilcoxon rank sum test showed that there was no significant difference between EHS++ and EHS+ (z = 0.970; *p* = 0.3300; Table 5).

### 4.3 Anxiety and depressive disorders

For anxiety and depressive symptomatology, the non-parametric Kruskal-Wallis test was performed and significant differences were found between the four groups, for both anxiety (X^2^(3) = 19.635; *p* = 0.0002) and depression (X^2^(3) = 3.286; *p* = 0.0001; Table 5). The Dunn’s *post-hoc* comparison test with Holm corrections (Table 6) revealed for:

- Anxiety: one significant difference between EHS and nonEHS group (*p* < 0.001).
- Depression: significant differences between EHS group and nonEHS group (*p* < 0.001).

**Table 6:** Dunn’s pairwise comparison according to categorization into four groups (EHS self-assessment).

		EHS		EHS?		SNS	
		Coefficient	*p*	Coefficient	*p*	Coefficient	*p*
Absorption	EHS?	1.342	0.449				
	SNS	0.807	0.839	−0.583	0.840		
	nEHS	1.552	0.362	−0.161	0.436	0.545	0.586
Anxiety	EHS?	1.441	0.150				
	SNS	2.282	0.056	0.524	0.300		
	nEHS	4.425	**0.000**	1.910	0.112	1.516	0.194
Depression	EHS?	1.055	0.437				
	SNS	1.024	0.306	−0.165	0.435		
	nEHS	4.674	**0.000**	2.364	**0.036**	2.984	**0.007**
Risk perception	EHS?	2.513	**0.024**				
	SNS	3.571	**0.001**	0.572	0.284		
	nEHS	5.441	**0.000**	1.531	0.189	1.023	0.306
Avoidance strategies	EHS?	1.544	0.123				
	SNS	5.617	**0.000**	3.090	**0.003**		
	nEHS	6.869	**0.000**	3.575	**0.001**	0.172	0.432

The significant values in bold.

For depression, no significant difference was observed by sex (*z* = 1.90; *p* = 0.06) or by age category (X^2^(4) = 6.89; *p* = 0.14). The same conclusion was reached for anxiety, where no significant difference was observed either by gender (*z* = 1.32; *p* = 0.19) or by age category (X^2^(4) = 2.28; *p* = 0.68).

Using the criteria of Szemerszky et al. (5), the Wilcoxon rank sum test showed that there was no significant difference between EHS++ and EHS+ for depression, but there was one for anxiety (*z* = 2.245; *p* = 0.025; Table 5).

### 4.4 Risk perception and avoidance strategies

For risk perception and avoidance strategies, the non-parametric Kruskal-Wallis test was performed and significant differences were found for the four groups, for both risk perception (X^2^(3) = 31.431; *p* = 0.0001) and avoidance strategies (X^2^(3) = 47.611; *p* = 0.0001; Table 5). The Dunn’s *post-hoc* comparison test with Holm corrections (Table 6) revealed for:

- Risk perception: significant differences between the EHS group and the EHS? group (*p* = 0.024), the SNS group (*p* = 0.001) and the nonEHS group (*p* < 0.001).
- Avoidance strategies: significant differences between EHS group and the SNS group (*p* < 0.001) and the nonEHS group (*p* < 0.001).

For risk perception, no significant difference was observed by sex (*z* = 0.90; *p* = 0.37), but a difference appeared by age categories (X^2^(4) = 10.98; *p* = 0.027). For avoidance strategies, no significant difference was observed neither by sex (*z* = −1.11; *p* = 0.27) nor by age categories (X^2^(4) = 3.45; *p* = 0.49).

Using the criteria of Szemerszky et al. (5), the Wilcoxon rank sum test showed that there were significant differences between EHS++ and EHS+ for both risk perception (*z* = 2.567; *p* < 0.02) and avoidance strategies (*z* = 4.375; *p* < 0.001; Table 5).

### 4.5 Comparison between EHS and highly sensitive people

**Figure 1:**
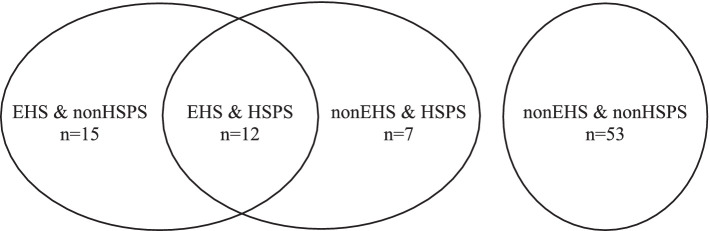
Comparison and distribution of participants in four groups: EHS & HSPS; EHS & nonHSPS, nonEHS & HSPS, nonEHS & nonHSPS.

We have already seen that EHS people are over-represented in the HSP group. At this stage, we want to check whether the EHS’ scores and the HSP’ scores on the different scales are similar or significantly different. To do this, we compared the results of four groups categorized as following: “EHS & HSPS,” “EHS & nonHSPS,” “nonEHS & HSPS” and “nonEHS & nonHSPS” (Figure 1; Table 7).

**Table 7:** Kruskal-Wallis tests comparing EHS & HSPS, EHS & nonHSPS, nonEHS & HSPS and nonEHS & nonHSPS.

		EHS & HSPS	EHS & nonHSPS	nonEHS & HSPS	nonEHS & nonHSPS	Chi2(3)	*p*
Absorption	Participants	12	12	7	51	13.792	**0.0032**
	Rank-sum	757.5	471.0	348.0	1826.5		
Anxiety	Participants	11	14	7	52	17.247	**0.0006**
	Rank-sum	735.5	677.5	336.0	1821.0		
Depression	Participants	12	14	7	51	15.751	**0.0013**
	Rank-sum	790.0	623.0	342.0	1815.0		
Risk perception	Participants	12	15	7	53	22.386	**0001**
	Rank-sum	778.0	913.0	309.0	1828.0		
Avoidance strategies	Participants	12	15	7	53	36.124	**0001**
	Rank-sum	736.0	1028.0	213.0	1851.0		

The significant values in bold.

The results showed that there were differences between the four groups for all five variables absorption (*p* = 0.0032), depression (*p* = 0.0013), anxiety (*p* = 0.0006), risk perception and avoidance strategies (*p* = 0.0001). Dunn’s post-hoc comparison tests with Holm’s corrections (Table 8) revealed several points:

- There were differences for all variables between the “EHS & HSPS” group and the “nonEHS & nonHSPS” group (*p* = 0.001).
- There was a difference for absorption between “EHS & HSPS” and “EHS-nonHSPS” (*p* = 0.035); in other words, being HSPS influenced the absorption score, but being EHS did not have any impact.
- The variable avoidance strategies showed that there was no significant difference between the two EHS groups (“EHS & HSPS” and “EHS & nonHSPS”); in other words, being HSPS or not did not influence the avoidance strategies. However, there were significant differences between these two groups and the two nonEHS groups (“nonEHS & HSPS” and “nonEHS & nonHSPS”; *p* < 0.007). This could be explained by the tendency of EHS sufferers to have found an external cause (EMF) for their symptoms and to try to reduce them by various strategies. However, the latter strategies are not necessary for HSPS-nonEHS people, as they do not associate their possible disorders with an external cause.

**Table 8:** Dunn’s pairwise comparison comparing EHS & HSPS, EHS & nonHSPS, nonEHS & HSPS and nonEHS & nonHSPS.

		EHS & HSPS		EHS & nonHSPS		nonEHS & HSPS	
		Coefficient	*p*	Coefficient	*p*	Coefficient	*p*
Absorption	EHS & nonHSPS	2.460	**0.035**				
	nonEHS & HSPS	1.186	0.353	−0.926	0.355		
	nonEHS & nonHSPS	3.581	**0.001**	0.451	0.326	1.451	0.294
Anxiety	EHS & nonHSPS	1.891	0.147				
	nonEHS & HSPS	1.609	0.161	0.035	0.486		
	nonEHS & nonHSPS	3.958	**0.000**	1.832	0.134	1.330	0.184
Depression	EHS & nonHSPS	2.231	0.064				
	nonEHS & HSPS	1.468	0.284	−0.387	0.349		
	nonEHS & nonHSPS	3.878	**0.000**	1.215	0.224	1.354	0.264
Risk perception	EHS & nonHSPS	0.406	0.343				
	nonEHS & HSPS	1.723	0.170	1.447	0.222		
	nonEHS & nonHSPS	3.759	**0.001**	3.572	**0.0009**	0.951	0.342
Avoidance strategies	EHS & nonHSPS	−0.826	0.409				
	nonEHS & HSPS	2.888	**0.006**	3.700	**0.001**		
	nonEHS & nonHSPS	3.671	**0.001**	5.107	**0.000**	−0.497	0.310

The significant values in bold.

## 5 Discussion

The aim of this study was to investigate the possible relationship between sensory processing sensitivity and electrohypersensitivity. Several hypotheses were tested and confirmed.

### 5.1 Electrohypersensitivity and sensory processing sensitivity

Our results confirm that people who self-report EHS report higher sensory processing sensitivity scores than others (hypothesis 1), and that they are significantly overrepresented in the group of people who can be considered highly sensitive according to Lionetti et al.’s classification (38) (hypothesis 3). They also scored particularly high on the LST, AES and CHA components (partially confirming our hypothesis 2). However, this was not the case for the EOE component. This seems to confirm that our EHS volunteers have a particularly low sensory threshold, which could explain their tendency to perceive internal and/or external stimuli more intensely and to take longer than others to return to their ‘acceptable’ level. This could be consistent with the hypotheses regarding the latency of symptom onset and recovery time expressed by EHS sufferers when exposed to such stimuli. This is also considered in the development of certain exposure study protocols designed with EHS subjects (67), which also highlights the need for and relevance of experiential knowledge in constructing exposure protocols and understanding the specificities of these subjects (74).

However, the measurement of sensory processing sensitivity using the only valid instrument to date (HSPS-FR) has already been questioned from a conceptual point of view (75). On the one hand, the weak convergent validity related to the low or even non-existent factorial correlations between the AES and the EOE raises questions. In this case, elements of an answer could be found in Horowitz’s interactionist model. In this model, the same educational environment can have different effects depending on the characteristics of the individual; and for the same characteristic, a facilitating or non-facilitating environment can influence the expression of a characteristic that generates adaptation/flexibility or vulnerability. On the other hand, the weak discriminant validity between the EOE and Big Five neuroticism (76) and its “self-consciousness” facet, or between the AES and “openness to experience” has been highlighted. However, the LST appears to be the only factor in sensory processing sensitivity that can potentially be disentangled from personality traits established in the Big Five (75). However, it is on this component and that of Controlled Harm Avoidance (CHA; the CHA items relate to the conscientious dimension of personality and/or their behavior) that the EHS seem to stand out, reporting higher scores than the others. It should be noted, however, that the CHA was not one of the components studied by Hellwig and Roth (75). Moreover, only a few authors have included this component in their model (43, 44, 52). However, this component raises new questions about the measurement of the HSPS. Indeed, it can be considered as a coping strategy that may also be associated with avoidance strategies, allowing individuals to avoid stimuli that may be perceived as unpleasant. In this sense, it is legitimate to ask whether this is a component of HSPS itself or a consequence of high sensitivity.

### 5.2 (Electro) High sensitivity and consequences

This study did not confirm previous findings on absorption (27) and we cannot confirm our fourth hypothesis. As a reminder, absorption corresponds to the ability to become deeply involved in a sensory and imaginary event while completely ignoring other stimuli. Since our results showed that the discriminant variable was not related to EHS status, but to being HSPS or non-HSPS, this raises an interesting point to answer the question of this article. If electrohypersensitivity is a specific form of high sensory processing sensitivity, then the previous results on absorption (27) could be the potential consequence of the high sensory processing sensitivity of the participants and not of being EHS.

EHS people also reported significantly higher scores than the other groups in terms of risk perception, avoidance strategies and anxiety-depressive symptoms (confirming globally our hypothesis 5). The absence of differences with SNS subjects (except for depression), whose non-specific symptoms of unknown origin, like those reported by EHS subjects, but not attributed to EMFs, seems quite logical, as these symptoms (whatever their origin) could be the consequence of this absorption.

These results confirm that there is a difference between EHS and non-EHS people, both in some of the characteristics that may explain their sensitivity, such as sensory processing sensitivity, and in the consequences they experience in terms of cognition (risk perception), emotions (anxiety and depression) and behavior (avoidance strategies). On the other hand, the scores of EHS people (absorption, anxiety, depression, risk perception and avoidance strategies) are significantly different from those of people who are neither EHS nor HSPS. With regard to avoidance strategies, and in contrast to absorption, the discriminant variable is EHS, as there was also a significant difference with the non-EHS-HSPS group. In this study, avoidance strategies are not the result of a personality trait (HSPS or absorption), but of the self-assessment as EHS people, i.e., as people suffering from symptoms related to external causes over which they believe they have some control, unfortunately ineffective, by avoiding electromagnetic sources. This seems to indicate that EHS people share certain characteristics with highly sensitive people, suggesting that EHS people may be highly sensitive like the others, but that the expression of this sensitivity is part of a different socio-cognitive logic and a different interpretation of the potential causes of this sensitivity. Furthermore, it was not possible to differentiate between EHS and SNS people, except for avoidance strategies. The same explanation can be used. And it would be interesting to develop more studies aimed at comparing these two groups, whether they are highly sensitive or not. Indeed, they seem to share at least absorption as a temperamental trait, and some characteristics such as anxiety and depression, which could be the consequence of their health status, regardless of its attributed cause.

Thus, the difference in terms of avoidance strategies seems to be explained by the major difference between EHS, SNS, or HSP. Electrosensitive people believe that EMFs, which are an external cause, are at the root of their symptoms. They may therefore try to reduce their symptoms through various strategies. However, these strategies are not necessary for SNS people, or highly sensitive people because they do not associate their possible disorders with an external cause, and perhaps they do not even *a priori* associate them with a cause outside their sensitivity. This difference in explanation is therefore based on a dichotomy between externality and internality: by associating their symptoms with an external agent (EMF in particular), electrosensitive people find a meaning that can explain their deteriorated state of health. This worsened state of health is also quite typical of highly sensitive people in general (48). It should be noted, however, that at this stage this study offers only exploratory elements that should be further investigated.

### 5.3 Qualifying electro-hypersensitivity

Apart from responding to the problem highlighted by Anses (1) of studying the influence of dispositional variables such as sensory processing sensitivity, the originality of this study lies in the results that highlight the relevance of classifying EHS individuals according to several questions, as recommended by Szemerszky et al. (5). In fact, this classification proved to be particularly effective for studying the influence of EHS on the participants’ scores in terms of risk perception, the avoidance strategies they tend to use and the anxiety symptoms they report, as well as the tendency we can observe for depressive symptomatology. These results generally support our hypotheses 6 and 7. For the traits “HSPS” and “absorption,” this has not yet been a relevant distinction. Overall, however, these results are surprising, not to say contradictory. Indeed, we might expect that people who use more avoidance strategies on a daily basis than others would report fewer symptoms as a result of these strategies. It seems, therefore, that these strategies are ineffective, confirming results already presented in the literature (77). Overall, this study provides some answers to the debate on the relationship between EHS and sensory processing sensitivity. It opens up interesting perspectives because our results, although exploratory at this stage, seem to testify to the existence of a relationship between the two. If EHS is a specific expression of high sensory processing sensitivity, it seems to be characterized by the tendency of EHS sufferers to explain the symptoms associated with their sensitivity in terms of the effects of electromagnetic waves. However, one of the major issues at this stage seems to be the well-established attributional processes of EHS sufferers with regard to the cause associated with their disorders. In fact, this process seems to leave no room for any alternative that could provide better medical and clinical support for those affected.

However, high sensory processing sensitivity may also be associated with a degree of empowerment and coping. Since many avoidance strategies of people with HSPS are ineffective and lead to social isolation, absenteeism from work, etc., it might be interesting to help both groups to develop effective strategies for controlling and avoiding distress by teaching them to identify problematic situations to avoid them more effectively. The role of sensory processing sensitivity in anxiety is well established [e.g., (45)], as is the tendency of people with EHS to report anxiety. Anxiety tends to revolve around future events, in contrast to depressive disorders, which often focus more on the present. By helping highly sensitive people to fear the future less through psychoeducational training, they may also learn to better control the potential annoyance of intrusive stimuli and thoughts that the future can evoke. Another hypothesis worth investigating is that highly sensitive individuals who undergo this type of psychoeducational training may subsequently score higher on the CHA component, which could then act as a protective factor against anxiety, whose scores could then be reduced accordingly. Finally, there could be another possible relationship between EHS and HSPS through health anxiety. The latter is defined as the fear of being affected by, or contracting and developing, a serious and/or chronic illness (78). Health anxiety is a predictor of EHS-related symptoms and is also associated with sensory processing sensitivity (79). It may therefore be interesting to investigate this point in more detail, as well as differences between the groups in terms of their personality traits, their emotions, or the behavioral or health consequences of their traits.

### 5.4 Limitations and perspectives

The results of this study are not without limitations, starting with the small size of the population (*n* = 100), which makes it difficult to generalize the results and conclusions. It should also be mentioned that the study is based on self-report questionnaires, which may introduce bias. The small, non-representative sample and the grouping based on self-assessment can be criticized. Nevertheless, they represent a first step in investigating the relationship between high sensitivity and electromagnetic sensitivity. Note the high proportion of males in our sample, which is rare enough in this type of study to be worth highlighting. In addition, the context of the study (during the Covid19 period) may have influenced the participants and their responses to the different scales. It would therefore be interesting to consider replicating this study in a more favorable context, with more participants in each group. Furthermore, our results may be partly explained by the overlap between some of the two groups (EHS and HSPS), as we have seen that highly sensitive people were over-represented in the EHS group. We are aware of the methodological limitations of the approach presented here. However, although there is room for methodological improvement, we believe that these results provide interesting research.

## Data Availability

The dataset analysed for this study is available online under simple request in the Zenodo repository: https://zenodo.org/records/14887029.
